# Implications of Public Disclosure of Personal Information in a Mobile Alert App for People Living With Dementia Who Go Missing: Qualitative Descriptive Study

**DOI:** 10.2196/64847

**Published:** 2025-02-07

**Authors:** Adebusola Adekoya, Christine Daum, Noelannah Neubauer, Antonio Miguel-Cruz, Lili Liu

**Affiliations:** 1 School of Public Health Sciences, Faculty of Health University of Waterloo Waterloo, ON Canada; 2 Department of Occupational Therapy, Faculty of Rehabilitation Medicine University of Alberta Edmonton, AB Canada

**Keywords:** alert systems, technology, missing persons, dementia, autonomy, privacy, stigmatization, consent

## Abstract

**Background:**

People living with dementia are at risk of getting lost and going missing due to memory loss, confusion, and disorientation. Missing person incidents involving people living with dementia are increasing. Alert systems such as Community ASAP can promote community engagement in locating missing persons with dementia and aid in search and rescue efforts. However, the implications of public disclosure of personal information such as name, age, sex, and physical description within such alert systems have yet to be explored.

**Objective:**

This study aimed to identify and discuss the implications of public disclosure of personal information in Community ASAP for people living with dementia at risk of going missing.

**Methods:**

This study used a qualitative descriptive research design drawing from naturalistic inquiry. A total of 19 participants including people living with dementia, care partners, first responders, and service providers were recruited from Ontario, Alberta, and British Columbia, Canada. Semistructured interviews were used to explore participants’ perspectives on the perceived implications of the release of personal information when using Community ASAP. NVivo (version 12) was used to manage data, and conventional content analysis was conducted to identify key themes of the implications of public disclosure of personal information in Community ASAP.

**Results:**

In total, 10/19 (53%) of the participants were women and 9/19 (47%) were men. Of the 19 participants, 3 (16%) were people living with dementia, 5 (26%) were care partners, 4 (21%) were first responders, and 7 (37%) were service providers. In total, 4 key themes were identified as implications of public disclosure of personal information in Community ASAP: *right to autonomy*, *safety versus privacy*, *informed and knowledgeable consent*, and *stigmatization.* Participants discussed how the public disclosure of personal information in Community ASAP could undermine a person’s choice not to be found and contribute to stigmatization. Participants emphasized a need to balance safety and privacy concerns. Informed and knowledgeable consent is important when using an alert system to locate missing persons with dementia.

**Conclusions:**

Community ASAP can promote community engagement in locating missing persons with dementia. However, the public disclosure of personal information in alert systems has implications. Users’ right to autonomy, a balance between safety and privacy, informed and knowledgeable consent, and risks of stigmatization are perceived impacts of disclosure of personal information in alert systems.

## Introduction

### Background

Dementia is an increasing health problem affecting >55 million people worldwide [1]. People living with dementia are at risk of getting lost and going missing due to memory loss, confusion, and disorientation [2]. Missing person incidents involving people living with dementia have been increasing [3]. Missing persons with dementia are at risk of being exposed to injuries and death. If not found within 24 hours, half of missing persons with dementia will sustain serious injuries or be found deceased [4]. It is crucial to engage the community in locating missing persons and not view missing person incidents solely as a family issue [5]. This inclusive approach provides extra eyes on the ground. Strategies such as alert systems promote community engagement by encouraging community members to be on the lookout for missing persons. This, in turn, aids in search and rescue efforts and minimizes the risks associated with going missing [6].

Alert systems such as the United States’ publicly funded Silver Alert program disseminate information about missing persons with dementia or other cognitive disabilities to the public through media broadcasts (eg, commercial radio and television stations) and electronic billboards [6]. The Silver Alert system has been implemented in all but 5 states [6]. However, there are limitations to these programs. The process of issuing Silver Alerts and the duration differ across states, and media broadcasts may reach fewer people due to an increase in the use of mobile devices to access information. Furthermore, there are concerns about declining media sensitivity due to alert fatigue [7].

In Canada, when a person living with dementia goes missing, police services alert the public about a missing person using social media such as Facebook and X (formerly Twitter) and not an emergency alert system such as an Amber alert [8]. Amber alerts notify the public about missing abducted children using Alert Ready. Alert Ready is Canada’s emergency alert system that sends critical alerts about hazards or impending dangers (eg, floods, tornados, chemical spills, and fires) to Canadians through radio, satellite television, and compatible wireless devices [9]. As missing person incidents involving people living with dementia are more frequent than missing children and not typically related to a crime, the use of the Alert Ready system would not be appropriate [8]. Furthermore, the use of the Alert Ready system can desensitize the public to alerts and cause alert fatigue, which already exists with Amber alerts.

Ideally, programs that mitigate risks of going missing among people living with dementia would engage local communities but minimize alert fatigue and be location specific. For example, the community-led British Columbia Silver Alert program notifies community subscribers about missing persons with dementia, cognitive impairment, and autism using channels such as social media (eg, Facebook and X) and via email or SMS text message [10]. Interest in community alert systems in other Canadian provinces is growing, as evidenced by a recent national petition; amendments to Missing Persons (Silver Alert) Acts in Alberta, Manitoba, and Ontario [11-13]; and the launch of Silver Alert pilot projects in Quebec [14].

To date, a localized alert system based on volunteer subscription does not exist. To this end, our team developed a localized area alert app called Community ASAP, the first of its kind in Canada, with representatives of end users, including people living with dementia, care partners, and community organizations [3]. Community ASAP allows community volunteers, local businesses, and police services to work together to locate missing persons with cognitive impairment and is available as a mobile app. As a requirement in Canada, a Community ASAP coordinator from police services would initiate an alert when a person living with dementia is reported missing. Community volunteers who register with Community ASAP receive alerts about missing persons based on their geographic preferences. The alert includes a link to the missing individual’s personal information, such as their first and last names, nickname, age, physical description (eg, height, weight, eye color, and hair color), mobile number (if any), and locations they typically visit [3].

Community ASAP and other public alert systems such as Silver Alerts and Amber alerts are designed to enhance public awareness of vulnerable missing persons, but they differ in their scope and approaches. Community ASAP is a subscription-based alert system that requires community engagement and provides real-time updates and geofencing features. For example, volunteers receive missing person alerts based on their geographic preferences, allowing them to choose up to 5 locations, such as home and work addresses, and set a radius (1, 3, 6, 12, or 25 km) for each location [3]. However, its effectiveness depends on widespread app adoption and access to mobile devices. In contrast, Silver Alerts and Amber alerts are government funded or community funded and broadcast information about missing persons through large-scale public channels such as highway signs, radio, and television, reaching a wider audience [6,15]. The activation of Community ASAP in Canada and public alert systems in the United States rely on police verification, which means that alerts are not triggered immediately when a missing person incident occurs [3,6,16].

This study was a part of the development and evaluation of the accuracy and usability of Community ASAP [3]. During the development of Community ASAP, we identified the need to understand how the rights of people living with dementia to self-determination and privacy are respected while using the alert system. The use of alert systems has raised ethical concerns about the privacy of people living with dementia [6,15]. Furthermore, there is limited knowledge about concerns related to using alert systems to locate missing persons with dementia [15], particularly how the release of personal information to the public may impinge on the human rights of vulnerable older adults [17].

### Objectives

The purpose of this study was to identify and discuss the implications of public disclosure of personal information in an alert system called Community ASAP for people living with dementia at risk of going missing.

## Methods

### Research Design

This study used a qualitative descriptive research design drawing from naturalistic inquiry, which allows a phenomenon to be studied in its natural state [18]. This method is appropriate when the intent of the study is to provide a basic description and summary of people’s experiences [19,20].

### Recruitment

Purposeful sampling [21] was used to select participants who had professional and personal experiences on the topic to best provide insights into concerns that could arise from the release of information via Community ASAP. Specific strategies included intensity [21] and snowball [22] sampling. Intensity sampling was used to sample information-rich cases. In snowball sampling, participants helped share study information with other prospective participants whom they believed had knowledge or experience with the topic of interest. These strategies were chosen for practical reasons, including the challenges of selecting potentially difficult-to-access or vulnerable groups, such as people living with dementia. We aimed to include the perspectives of people living with dementia, who are often underrepresented in research. Studies have shown the importance of involving people living with dementia in research, highlighting their ability to provide reliable self-report information, personal perspectives, and valuable insights [23-25]. Inclusion of people living with dementia in this study was crucial to understanding their individual experiences with an alert system and enhancing their safety and well-being while using this program.

Participants (people living with dementia, care partners, first responders, and service providers) were recruited and identified via email or face-to-face conversations through our research team’s existing professional networks serving people living with dementia, such as Alzheimer societies, dementia advocacy organizations, and first responders. Thus, some of the participants were known to the research team, and others were not. None of the participants were family members or friends of or had personal relationships with the researchers. All prospective participants who were invited to take part in the study enrolled. Participants had lived or professional experience with dementia and the use of technologies to manage the risk of going missing or responding to missing person incidents. During recruitment, participants were asked to self-identify their experience and knowledge of technologies. All participants had some level of familiarity or experience with technology. We did not inquire about the extent or specific type of their experience.

We selected participants from across 3 Canadian provinces (Ontario, Alberta, and British Columbia) where our professional networks were located and from 4 stakeholder groups: people living with dementia, care partners, service providers (eg, social workers, dementia educators, representatives from vulnerable person registries, and support workers), and first responders (eg, police and search and rescue managers). Inclusion criteria were to (1) be aged ≥18 years, (2) speak English, and (3) have lived or professional experience with or knowledge of the use of technologies to manage the risk of going missing or responding to missing person incidents. Exclusion criteria were having no understanding about technologies to manage the risk of going missing, inability to articulate perspectives due to impaired cognitive abilities, and severe visual or hearing limitations that could not be corrected with the use of an assistive device.

### Ethical Considerations

Ethics approval was received from the University of Alberta Research Ethics Board (Pro00078537). All participants gave written informed consent prior to the study, were told that their participation is voluntary, and were informed of their right to withdraw at any time. People living with mild cognitive impairment or mild dementia who had the ability to consent as identified by our professional networks were invited to participate in this study. We used the teach-back method [26] to ensure that these participants had cognitive ability to engage in one-on-one interviews with research team members. The person living with dementia was asked an open-ended question about what they had read in the information letter to ensure that they understood the procedure, risks, and what to do should they wish to withdraw from the study. Transcripts were cleaned for accuracy and deidentified to maintain anonymity. Participants did not receive an honorarium.

### Data Collection

Semistructured interviews [27] were conducted with each participant in person, via phone, or through Zoom (Zoom Video Communications) videoconference. The purpose of the interviews was to understand participant perspectives on the implications of public disclosure of personal information in alert systems. Each person selected a preferred mode of participation based on their geographical location and other time commitments. In total, we interviewed 19 stakeholders (n=16, 84% individual interviews and 1 group interview with n=3, 16% of the participants at their request). No other individuals (ie, care recipients or care partners) were present during the interviews with people living with dementia as these individuals had the ability to consent and respond to questions. Before the interview began, the researcher introduced herself, the overarching study [3] in which the current project was embedded, and the purpose and procedures, and written informed consent was obtained. An interview guide (Textbox 1) that contained 5 open-ended questions was used to elicit participants’ perspectives on the possible implications of the release of information through Community ASAP. Probes were used to elaborate on participant responses and clarify meaning [22]. The interviews were conducted by female research team members (NN and CD) with backgrounds in occupational therapy, experience with qualitative research methods, and PhDs in rehabilitation science. Each participant was interviewed once, and the interviews were approximately 30 to 90 minutes in length. Interviewers documented their observations and any interruptions in field notes, and the interviews were digitally recorded and transcribed verbatim. Transcripts were not returned to participants as we did not use a member-checking approach to review the transcripts. NVivo (version 12; Lumivero) was used to manage the data.

**Interview guide questions.**

**Questions**

- From your perspective as a (person with dementia or caregiver of a person with dementia, service provider, first responder, expert in ethics, or the law), what ethical concerns are associated with release of personal information in the C-ASAP system?
- From your perspective as a (person with dementia or caregiver of a person with dementia, service provider, first responder, expert in ethics, or the law), what legal concerns are associated with release of personal information in the C-ASAP system?
- How does the release of personal information in the proposed C-ASAP system compare with other registries such as MedicAlert Safely Home and various Vulnerable Persons Registries?
- What are the implications of having a missing person’s name released to the public (and thus a part of the public record)? What is the balance (or tipping point) between privacy and safety?
- [For group interviews involving representatives from agencies that collect information about vulnerable persons and store them on registries]: What agreements are in place between the vulnerable person or their family and the agency upon signing on with a particular registry?

### Data Analysis

Conventional content analysis [28] guided our analytic approach. A thematic approach is preferred when there is limited existing theory or research literature on a phenomenon, allowing for the identification of specific meanings and determination of appropriate categories and themes. A female doctoral candidate in public health sciences (AA) with a background in nursing and qualitative research methods analyzed the data. She immersed herself in the data by listening to the recordings while reading the transcripts and identifying initial reflections. Next, she coded the transcripts using keywords. Codes were described, and similar codes were grouped and refined to create categories. As new codes were generated, they were added to the framework, and transcripts that had been previously coded were updated to reflect hierarchy. Category descriptions were generated to describe their contents, and themes that represented the categories were created inductively. Analysis continued until saturation of the data was reached.

Data analysis was an iterative process. The coding hierarchy, categories, and themes were reviewed, scrutinized, and confirmed by the analyst and the 2 team members who conducted the interviews; that is, we used peer debriefing as a trustworthiness strategy [29]. This helped us improve clarity and the internal and external homogeneity of codes and categories [30] and consider alternative interpretations and explanations. Thus, the coding hierarchy and main themes were refined repeatedly, thereby enhancing credibility. Our process is consistent with the approach by Morse et al [31] to verification in which transcripts, codes, categories, and themes are rechecked.

## Results

### Overview

A total of 19 participants (n=10, 53% women and n=9, 47% men) were interviewed. Of these 19 participants, 3 (16%) were people living with dementia, 5 (26%) were care partners, 4 (21%) were first responders (search and rescue and police officers), and 7 (37%) were service providers. Participants were predominantly White individuals (17/19, 89%), and the remainder were Asian individuals (Korean and Filipino; 2/19, 11%; Table 1). All participants had some level of familiarity or experience with technology. In total, 58% (11/19) of the participants (first responders and service providers) had professional experience with technology to manage or respond to missing person incidents, such as alert systems, MedicAlert, and locator devices (GPS devices and Project Lifesaver). The remaining participants (people living with dementia and care partners; 8/19, 42%) had lived experience or familiarity these technologies.

**Table 1:** Study participants.

		Care partners (n=5), n (%)	First responders (n=4), n (%)	People living with dementia (n=3), n (%)	Service providers (n=7), n (%)
**Sex**					
	Male	4 (80)	4 (100)	1 (33)	0 (0)
	Female	1 (20)	0 (0)	2 (67)	7 (100)
**Ethnicity**					
	Filipino	0 (0)	0 (0)	0 (0)	1 (14)
	Korean	1 (20)	0 (0)	0 (0)	0 (0)
	White	4 (80)	4 (100)	3 (100)	6 (86)
**Province**					
	Alberta	1 (20)	1 (25)	0 (0)	0 (0)
	British Columbia	1 (20)	1 (25)	1 (33)	2 (29)
	Ontario	3 (60)	2 (50)	2 (67)	5 (71)

### Thematic Findings

#### Overview

Four key themes represent the implications of public disclosure of personal information in Community ASAP for people living with dementia who have gone missing: (1) right to autonomy, (2) safety versus privacy, (3) informed and knowledgeable consent, and (4) stigmatization (Textbox 2).

**Themes with participant quotes.**

**Right to autonomy**

- “Depending on where the person is in their journey, what if they just wanted to be alone for a few days and then got this whole thing going on because someone decided that they’ve disappeared. That’s a legal issue for me.” [Person living with dementia 3, an experienced user of GPS devices who had previously been lost]
- “We locate a missing person, we let them know you’ve been reported missing, here’s the person who’s reported you missing...However, they do have a right to be missing so to speak.” [First responder 4, an experienced police officer with expertise in search and rescue]
- “Is that what they wanted when they were in the real self before dementia, there is an ethical issue there because you’re not honoring what the person wants in the moment.” [Service provider 3, a service provider who worked directly with people living with dementia providing education and counseling]

**Safety versus privacy**

- “The more eyes the better. I think if you’re missing, I don’t think privacy should be an issue.” [Person living with dementia 1, who had experience with MedicAlert]
- “I think that the tipping point is safety, and so that might be it is 30 to 20 degrees Celsius outside. It’s not as extreme situation as it is 30 or minus 25. So, all of these things need to be taken into account.” [Care partner 2, a family member who cared for a person living with dementia]
- “Some people aren’t as open to having other people or the general public know a person with dementia has been missing more around, that they have the condition, they have dementia.” [Service provider 6, a person with expertise in supporting the safety of vulnerable persons and who had experience with GPS locator devices for this population]
- “If they are making this phone call it’s urgent enough, it’s important enough that yes, we are willing to give up that right to this in order to find this person quickly.” [First responder 1, an experienced search and rescue member who also provided education about locator devices]

**Stigmatization**

- “For a lot of people, I worry about their faces and the local papers, this stuff. And that still makes them very vulnerable, because someone recognizes them after the fact.” [Person living with dementia 3, an experienced user of GPS devices who had previously been lost]
- “So, with my dad, we didn’t want anybody to know that he had Alzheimer’s. I think it’s because there may be a stigma of Alzheimer’s.” [Care partner 3, a family member of a person living with dementia who got lost]
- “So, it’s such a stigma, it’s such a huge issue that sometimes you might think that having an alert system would be really useful for the community to know how to interact or to keep their eye out for somebody with dementia in the community. But is a stigma sort of perpetuating.” [Service provider 7, a service provider who works directly with people living with dementia providing education and counseling]
- “The one thing we are always cognizant of in our media releases, is our management and our upper management are always cognizant of the...stigma.” [First responder 3, a police officer with experience with missing persons]

**Informed and knowledgeable consent**

- “If the person is like me and can give consent, that would be OK...if I’ve already deteriorated, then I think it would be up to my family or my power of attorney because they know what my wishes are.” [Person living with dementia 2, a person who was previously lost]
- “Whenever possible, that person who is actually living with dementia, I certainly would advocate and support their involvement from the get-go. No matter what the timeframe, I think you need to be respectful and try to include their voice as much as possible, knowing that cognitively that could change at any time, and if it does then I would hope that the person has the best interests for that individual.” [Care partner 5, a family member of a person living with dementia who had experience with locator devices]
- “I think the idea of capacity and the ability to give informed consent is not an on/off button. And to always have that conversation as much as possible to the extent that it is possible for you and whether that’s through care partners. I think it’s really at the heart of it, really enabling that person to have the choice. But the challenge with that is that on one day the person might say ‘yeah I’m completely fine with it’ and then the next day they’re like ‘ugh, I’m not fine with it at all.’” [Service provider 7, a service provider who works directly with people living with dementia providing education and counseling]
- “Informed consent is the only consent that would certainly help alleviate some problems down the road, and I would think if the person can’t give consent, because of their mental capacity, hopefully the caregiver would have that Power of Attorney to be able to make that decision for them, on their behalf.” [First responder 2, an experienced police officer and search and rescue expert]

#### Right to Autonomy

This theme relates to concerns about how the release of personal information can compromise a person’s choice not to be found. Participants discussed the importance of respecting people’s autonomy and right to go missing without wanting to be found. People living with dementia also possess the rights of adults and have the right to be alone or go missing, whereas this does not apply to children. A participant (first responder 4) with many years of experience in search and rescue emphasized adults’ rights to go missing. He expressed the following:

When we locate a missing person, we let them know you’ve been reported missing, here’s the person who’s reported you missing...However, they do have a right to be missing so to speak.

Labeling intentionally missing persons as missing, especially those capable of decision-making, may raise ethical and legal concerns, as noted by some participants. This concern was further expanded by a participant living with dementia:

Depending on where the person is in their journey, what if they just wanted to be alone for a few days and then got this whole thing going on because someone decided that they’ve disappeared. That’s a legal issue for me. Like where in the journey it is decided that someone...I don’t even know how to explain that because like for me, it’s not uncommon for me, I mean now I always tell somebody just because that’s my life. I quite often go “Oh well I’m going to take off and go here for a couple of days.”

Person living with dementia 3, an experienced user of GPS devices who had previously been lost

Participants pointed out that individuals’ views on risk and outlook on life may change as their dementia progresses. Hence, it is crucial that the use of Community ASAP respects the preferences of those living with dementia. In addition, there might be a need to re-evaluate personal information disclosure preferences as dementia progresses. A service provider highlighted the importance of honoring the current wishes of people living with dementia when using Community ASAP:

Somebody’s idea about what they consent to and what they want when they’re first diagnosed might be different than what they want later on, and what’s the record of truth. Is that what they wanted when they were in the real self before dementia, there is an ethical issue there because you’re not honoring what the person wants in the moment.

Service provider 3, a service provider who worked directly with people living with dementia providing education and counseling

#### Safety Versus Privacy

This theme highlights concerns related to the balance between safety and privacy, acknowledging the diverse perspectives on how to maintain this balance for missing persons. While Community ASAP serves as a safety net, offering peace of mind to people living with dementia and their care partners, it also facilitates a sense of independence. However, some participants expressed concerns about privacy when it comes to sharing their personal information publicly. For many, safety was the tipping point as finding the missing person alive and minimizing the risks of harm is paramount. A care partner emphasized the urgency of finding missing persons promptly, especially in severe weather conditions in Canada:

I think that the tipping point is safety, and so that might be it is 30 to 20 degrees Celsius outside. It’s not as extreme situation as it is 30 or minus 25. So, all of these things need to be taken into account.

Care partner 2, a family member who cared for a person living with dementia

Privacy concerns are influenced by culture as cultural beliefs play a key role in how individuals view privacy, particularly regarding the disclosure of certain information to the public. For instance, a Korean participant (care partner 3, a family member of a person living with dementia who got lost) shared cultural beliefs about having a “conservative background” and keeping his father’s dementia diagnosis confidential within the family and refraining from sharing it with outsiders.

There are concerns regarding the permanence of personal information on the internet. Details such as a missing person’s name, age, photo, and physical description shared with the public might linger on the internet even after the person is located. Some participants expressed unease about their personal information being public posthumously.

Participants also emphasized the importance of only sharing essential information, such as the person’s name, age, photo, and physical description. While some information is necessary for community members (volunteers) to identify a missing individual, disclosing medical information (eg, cognitive impairment) could heighten the risks of fraud, identity theft, and abuse. One participant raised concerns about potential exploitation of people living with dementia when using Community ASAP:

The risk is that the volunteers would prey upon this person if he/she is found and returned home, identified to have dementia. They are already vulnerable to scammers, to people who would be able to redirect them and take advantage of them.

Service provider 1, a service provider who worked directly with people living with dementia providing education and counseling

Some participants expressed that a dementia diagnosis could make individuals vulnerable to exploitation regardless of whether their personal information is shared via an alert system such as Community ASAP. One participant (person living with dementia 1) stressed the significance of not disclosing banking details to the public:

You’re not going to give out any information regarding bank accounts. If people want to take advantage of you, they’re gonna do it anyways.

#### Stigmatization

This theme discusses concerns about how the use of Community ASAP could lead to stigma for people living with dementia. People living with dementia may experience stigma due to their dementia diagnosis and assumptions about their abilities and behaviors. People may assume that individuals living with dementia do not have the capacity to make decisions for themselves. Therefore, sharing personal information such as a cognitive impairment or dementia diagnosis publicly could heighten the existing risk of stigmatization for people living with dementia. A police officer (first responder 3) with experience with missing persons expressed the following:

*The one thing we are always cognizant of in our media releases, is our management and our upper management are always cognizant of the...stigma.*

A service provider also explained the stigma associated with using alert systems:

So, it’s such a stigma, it’s such a huge issue that sometimes you might think that having an alert system would be really useful for the community to know how to interact or to keep their eye out for somebody with dementia in the community. But is a stigma sort of perpetuating.

Service provider 7, a service provider who works directly with people living with dementia providing education and counseling

Participants pointed out that the public may not understand how to approach people living with dementia or communicate with them, hence the need to address dementia-related stigma and misconceptions through public education and awareness campaigns. It was also noted that service providers and first responders should receive adequate training on recognizing, communicating with, and responding to missing persons with dementia. A service provider discussed the importance of using nonstigmatizing language when interacting with missing persons with dementia:

I think one of the issues we may see is how responsive behaviors are presented. So, if there are triggers, because I know it is important to communicate that to the community, like you know do not touch this person because they might respond in this way. How can we train coordinators to use language that is going to be non-stigmatizing but still express the nature of that behavior.

Service provider 1, a service provider who worked directly with people living with dementia providing education and counseling

#### Informed and Knowledgeable Consent

This theme focuses on concerns related to ensuring individuals’ informed and knowledgeable consent when using Community ASAP. Participants highlighted the importance of people living with dementia having the capacity to understand the implications of an alert system, its objectives, and the reasons behind collecting and sharing their personal data. They emphasized the necessity of providing sufficient information about Community ASAP to those living with dementia and respecting their autonomy in decision-making. However, dementia can affect an individual’s ability to process and understand information adequately, leading to challenges in decision-making regarding alert systems, which does not always align with a simple *present or absent* perspective. This concern was elaborated on by a service provider:

I think the idea of capacity and the ability to give informed consent is not an on/off button. And to always have that conversation as much as possible to the extent that it is possible for you and whether that’s through care partners. I think it’s really at the heart of it, really enabling that person to have the choice. But the challenge with that is that on one day the person might say “yeah I’m completely fine with it” and then the next day they’re like “ugh, I’m not fine with it at all.”

Service provider 7, a service provider who worked directly with people living with dementia providing education and counseling

For individuals with limited decision-making capacity, service providers should seek consent from their proxies or substitute decision makers (eg, relatives) and make decisions in their best interests. Participants emphasized the importance of a respectful decision-making process that considers the evolving wishes and preferences of individuals with dementia as the condition advances. A first responder discussed the significance of informed consent and ensuring that decisions made on behalf of an individual living with dementia align with their preferences:

Informed consent is the only consent and that would certainly help alleviate some problems down the road, and I would think if the person can’t give consent, because of their mental capacity, hopefully the caregiver would have that Power of Attorney to be able to make that decision for them, on their behalf. What’s the end goal? The end goal is to try and help that person the best you can, and if they are in a position that is maybe not good for them, at least hopefully we put enough checkmarks in place that they’ll come out okay, and that’s what you have to look at.

First responder 2, an experienced police officer and search and rescue expert

The need to involve people living with dementia in the decision-making process regarding the use of Community ASAP was discussed by participants. It was emphasized that involving the person with dementia in decisions at an early stage is crucial as dementia progression can greatly impact decision-making capacity. Care partner 5, a family member of a person living with dementia who had experience with locator devices, expressed the following:

You need to be respectful and try to include their voice as much as possible, knowing that cognitively that could change at any time, and if it does...I would hope that the person has the best interests for that individual.

## Discussion

### Principal Findings

Our study aimed to identify the implications of public disclosure of personal information in an alert system called Community ASAP for people living with dementia who are at risk of going missing. The implications were discussed under 4 key themes: *right to autonomy*, *safety versus privacy*, *stigmatization*, and *informed and knowledgeable consent.*

Community ASAP differs from public alert systems for missing persons with dementia such as Silver Alerts. While Community ASAP is a subscription-based, localized alert system that provides real-time updates and geofencing features, Silver Alerts can be government funded or community funded and use large-scale channels such as highway signs and media to reach a wider audience [3,6]. Given the limited research on subscription-based, localized alert systems for missing persons with dementia, our references drew primarily from studies on Silver Alerts.

Participants raised concerns about respecting individuals’ right to autonomy when using Community ASAP. The literature emphasizes the importance of honoring the autonomy of people living with dementia in alert systems [17,32]. Autonomy, or self-determination, is a fundamental human right tied to a person’s capacity to assess options, weigh risks, communicate choices, and act accordingly [33,34]. Unlike children requiring guardianship, adults with dementia, even those with appointed guardians, retain some decision-making capacity [33].

People living with dementia may go missing intentionally to avoid disclosing their location or unintentionally due to disorientation or difficulty recognizing familiar places [16,35]. Respecting the right to go missing intentionally is controversial as risk is often viewed negatively and people living with dementia are considered a vulnerable group needing protection [36,37]. The use of technology to locate missing persons raises ethical concerns about balancing autonomy and privacy with safety [6,17,34]. For example, dementia-related wandering is typically seen as “risky” due to potential harm, but it can also represent autonomy and agency and even provide health benefits as a form of exercise [38]. In care settings, health care providers may monitor “wandering” due to ethical responsibilities and potential litigation fears [39]. However, disclosing personal information can violate a person’s autonomy [6]. It is crucial to balance safety with respect for autonomy, necessitating open discussions between people living with dementia and their care partners or health care professionals [39]. These conversations should focus on the person’s values, care preferences, and ways to support their choices while living with risks.

The public disclosure of personal information in Community ASAP also raises concerns about balancing safety and privacy. While safety focuses on locating missing persons and minimizing risks of harm, privacy emphasizes the right to choose what information is shared with others [33]. Participants expressed that disclosing details such as dementia diagnoses could violate privacy rights. Similarly, Wasser and Fox [32] found in their analysis of Silver Alerts that such systems can unintentionally infringe on the privacy rights of individuals with dementia. In addition, cultural beliefs influence perceptions of dementia [40] and privacy as some cultures view such diagnoses as private. Understanding these cultural factors is essential when using alert systems while ensuring the privacy of people living with dementia.

For most participants, the safety of missing persons outweighs privacy rights. The literature shows that care partners often prioritize safety over privacy concerns [15,16]. Alert systems can serve as a safety net for people living with dementia, providing comfort and independence while easing care partners’ worries. Balancing safety and privacy requires understanding individual preferences and the perceptions of risk. Disclosure of personal information may be necessary for safety, but technology can also create a false sense of security if it fails [8,16,33]. To reduce the risk of individuals going missing, additional measures such as GPS devices and return home interviews should be used alongside alert systems [8]. Informed dialogue among people living with dementia, care partners, and health care professionals is essential, along with effective policies to address these concerns [6,16,33].

Public disclosure of personal information in Community ASAP can contribute to the stigmatization and abuse of people living with dementia. Stigma can lead to delays in seeking help, exclusion from social activities, and reduced quality of life [41,42]. Participants in this study emphasized the need for dementia education and staff training to address stigma and improve communication regarding missing persons. Community education is also crucial to raise awareness about the risks associated with alert systems [6,8]. Furthermore, a participatory approach in the design, development, and evaluation of alert systems is necessary [3].

People living with dementia are at heightened risk of abuse due to their diagnosis [43,44]. Publicly sharing information such as dementia diagnoses and home addresses can lead to financial exploitation and other crimes. For example, the United States’ Silver Alert may broadcast home addresses, license plates, and photographs of missing persons, unintentionally increasing risks of victimization or identity theft [7,16,17]. It is vital to limit the information shared in alert systems to what is necessary and implement safety measures such as using general locations instead of specific addresses and ensuring that sensitive data are controlled and removed promptly [3].

Another concern regarding the public disclosure of personal information in Community ASAP is informed and knowledgeable consent. Consent is considered knowledgeable if “the individual knows the purposes of the collection, use or disclosure and knows that they have the right to give, withhold or withdraw consent” [45]. Participants noted that people living with dementia may face challenges in providing informed consent for using alert systems, often due to assumptions about their capacity [6,17]. While dementia can impact decision-making capacity in some cases, individuals can still make decisions with support from family, friends, or health care professionals [33,46]. Assessment of decision-making capacity can be difficult as dementia-related cognitive fluctuations affect attention and memory [46,47]. A dementia diagnosis does not automatically imply incapacity [46], and capacity assessments should account for the person’s stage of dementia and be conducted during moments when they are experiencing minimal confusion [47]. Continuous consent should be sought as the condition progresses, and early discussions are encouraged to address future decision-making needs. For those requiring substitute decision makers, involving them in decisions respects autonomy and dignity. Regardless of dementia progression, individuals should be included in conversations about their health and the use of alert systems, ensuring that their voices are considered in the design and implementation of these technologies.

### Strengths and Limitations

While there is research on public alert systems such as Silver Alerts, little is known about the implications of public disclosure of personal information in subscription-based, localized alert systems. Our study addressed this gap by examining how Community ASAP may infringe on the rights of people living with dementia. Specifically, it highlights the need to understand and address end users’ concerns about disclosure of personal information, the right to autonomy, the balance between safety and privacy, and informed and knowledgeable consent. Including participants with lived and professional experience with dementia and technologies for managing risks of going missing provided diverse perspectives on a topic with limited existing research. The qualitative descriptive approach used in this study enabled an in-depth exploration of concerns associated with public disclosure of personal information in subscription-based, localized alert systems.

Community ASAP and this study have limitations. The subscription-based, localized design of Community ASAP and the small participant sample size for each category limit its generalizability. The subscription model may exclude lower-income users and create a self-selection bias, whereas the app’s reliance on a high density of community users may reduce its effectiveness in underengaged or rural areas. These factors may have influenced participant responses and perspectives. To enhance broader applicability, addressing barriers such as cost, accessibility, and adoption in diverse communities would be essential in future research.

In addition, it was unclear how much experience participants had with localized alert systems. Thus, the concept of the release of personal information in such alert systems was hypothetical. Participants’ perspectives may differ with real-world experience, and the abstract nature of the topic may have led to the generation of data that are somewhat limited compared to those that a more concrete topic would yield. Recruitment methods also presented a limitation, potentially privileging certain voices. Participants were predominantly White individuals, which does not represent the demographics of Canada. In addition, participants were recruited through the researchers’ networks, likely including individuals who were more vocal, confident, or willing to participate. This may have excluded underrepresented perspectives such as those from ethnic communities where alert systems might be viewed differently or where stigma around dementia is more significant.

### Conclusions

Localized alert systems such as Community ASAP differ from existing Silver Alert programs that push alerts to the public through the media. Instead, Community ASAP pushes alerts to targeted community members who volunteer to be extra eyes and ears on the lookout for missing persons with dementia. This can promote community engagement in locating missing persons with dementia. However, the public disclosure of personal information in alert systems has significant implications. Such disclosure can compromise an individual’s right to autonomy and privacy and lead to stigmatization. The balance of privacy rights with safety concerns presents a challenge. Informed and knowledgeable consent should be a fundamental part of using alert systems. Regardless of the stage of dementia, individuals living with the condition have a right to be included in conversations about their health, including the use of alert systems. An understanding of these implications paves the way to respecting the rights of people living with dementia at risk of going missing and improving their safety and well-being.
